# Author Correction: Pro-metastatic collagen lysyl hydroxylase dimer assemblies stabilized by Fe^2+^-binding

**DOI:** 10.1038/s41467-018-05295-1

**Published:** 2018-07-10

**Authors:** Hou-Fu Guo, Chi-Lin Tsai, Masahiko Terajima, Xiaochao Tan, Priyam Banerjee, Mitchell D. Miller, Xin Liu, Jiang Yu, Jovita Byemerwa, Sarah Alvarado, Tamer S. Kaoud, Kevin N. Dalby, Neus Bota-Rabassedas, Yulong Chen, Mitsuo Yamauchi, John A. Tainer, George N. Phillips, Jonathan M. Kurie

**Affiliations:** 1 Department of Thoracic/Head and Neck Medical Oncology, The University of Texas MD Anderson Cancer Center, Houston, TX 77030 USA; 2 Department of Molecular and Cellular Oncology, The University of Texas MD Anderson Cancer Center, Houston, TX 77030 USA; 3 North Carolina Oral Health Institute, School of Dentistry, University of North Carolina at Chapel Hill, Chapel Hill, NC 27599 USA; 4 Department of Biosciences, Rice University, Houston, TX 77251 USA; 5 Division of Medicinal Chemistry, Targeted Therapeutic Drug Discovery and Development Program, College of Pharmacy, The University of Texas at Austin, Austin, TX 78712 USA; 6 Division of Chemical Biology & Medicinal Chemistry, College of Pharmacy, The University of Texas at Austin, Austin, TX 78712 USA; 7 Department of Medicinal Chemistry, Faculty of Pharmacy, Minia University, El-Minia 61519, Egypt

Correction to: *Nature Communications*; 10.1038/s41467-018-02859-z; published online 06 February 2018.

In the originally published version of this Article, financial support was not fully acknowledged. The PDF and HTML versions of the Article have now been corrected to also include support from the National Institutes of Health grant T32GM008280 to Sarah Alvarado.

