# Supplementary material for: Pro-metastatic collagen lysyl hydroxylase dimer assemblies stabilized by Fe2+-binding
Source: Nat Commun. 2018 Feb 6;9:512. doi: 10.1038/s41467-018-02859-z (PMC5802723; doi:10.1038/s41467-018-02859-z)
Supplement: Supplementary file 1 — Supplementary Information [file 41467_2018_2859_MOESM1_ESM.pdf]

Supplementary File:

Guo et al. “Pro-metastatic collagen lysyl hydroxylase dimer assemblies stabilized by Fe^{2+}–binding” (NCOMMS-17-24325A)

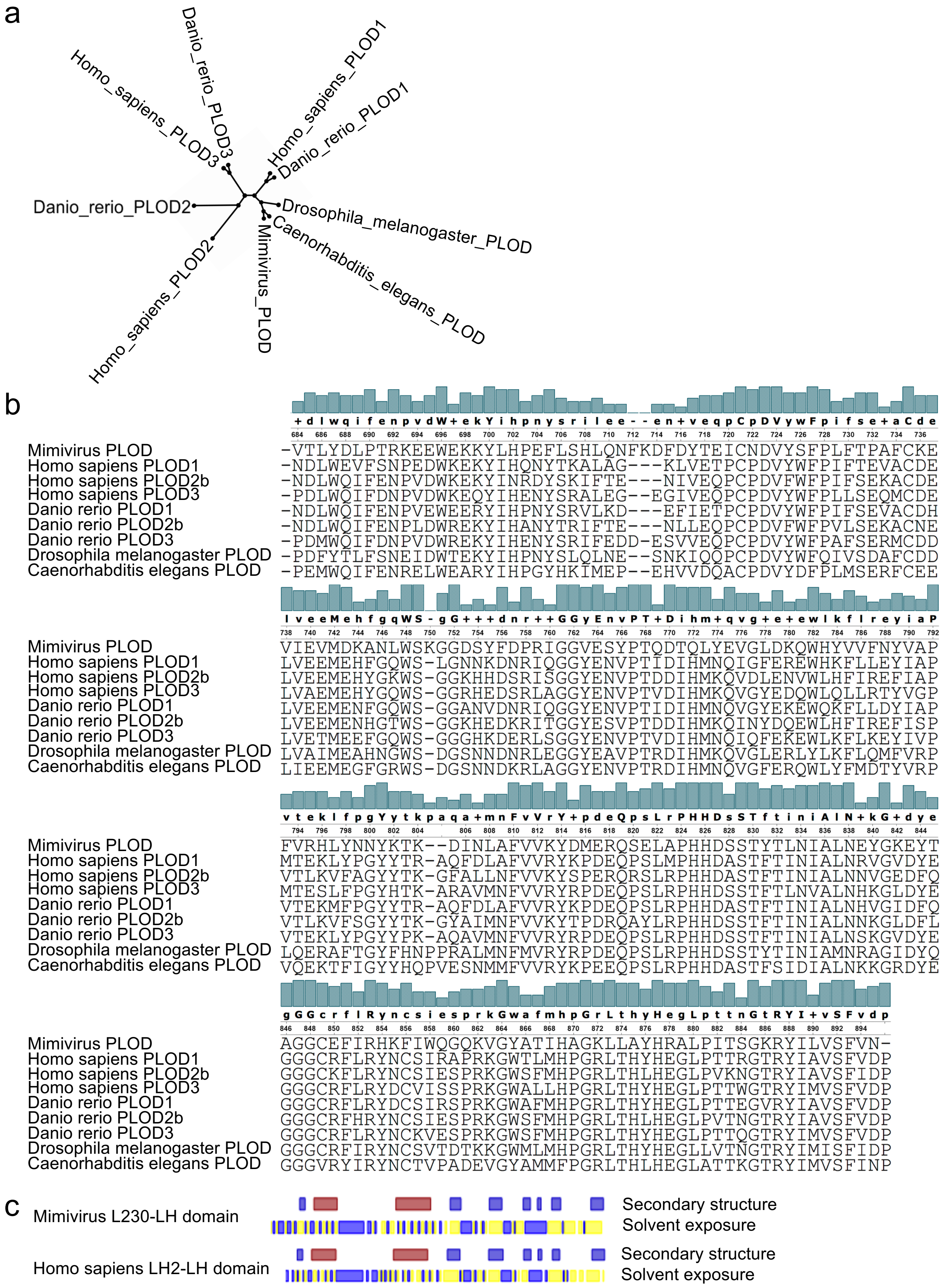

Supplementary Figure 1 │ Sequence alignment of L230 with invertebrate and vertebrate LHs. a and b, A cladogram of LHs (a) was generated based on the amino acid sequence alignment (b) of collagen LHs from Homo sapiens, Danio rerio, Drosophila melanogaster, Caenorhabditis elegans and Mimivirus using a CLUSTALW server (http://www.genome.jp/tools-bin/clustalw). Amino acids are color-coded. The gene name, protein name and UniprotKB accession number of these LHs are shown in Supplementary Table 1. **c,** Predicted secondary structures (α helices, red; β sheets, blue) and solvent–exposed regions (exposed, blue; non-exposed, yellow) in L230 and human LHs using a prediction algorithm (www.predictprotein.org).

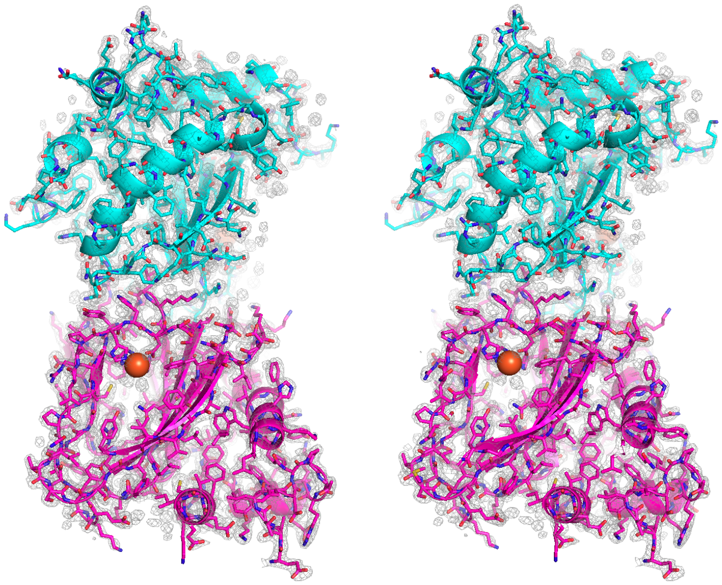

Supplementary Figure 2 │ Stereo view of electron density map ($2F_o$-F_c) of L230 LH domain. The contour level for map is 1.0σ.

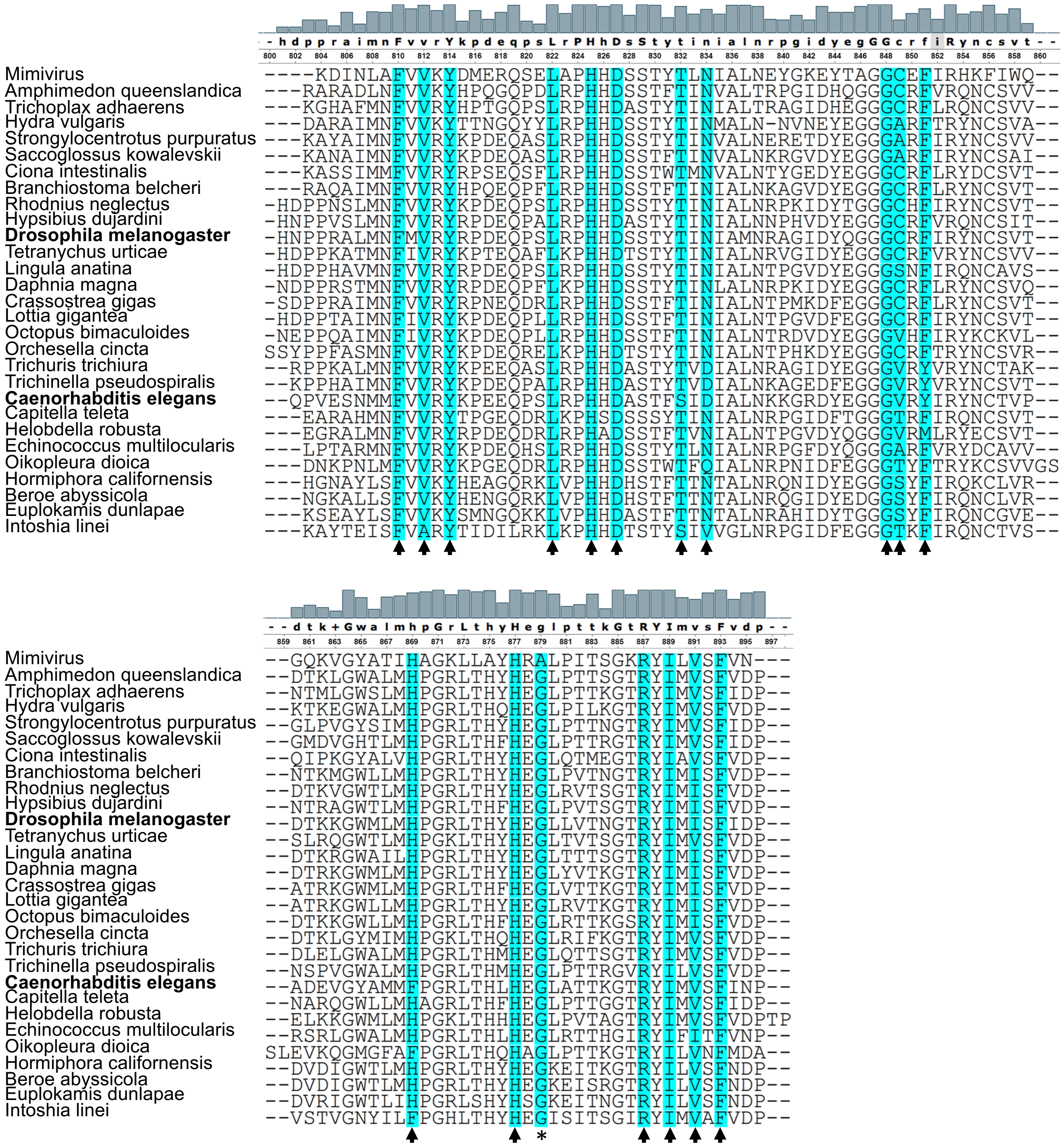

Supplementary Figure 3 │ Sequence alignment of L230 with invertebrate LHs. Residues with side chains facing the 2-OG binding pocket (cyan) that are conserved are indicated (arrows). Non-conserved A869 is indicated (asterisk). Species and NCBI GenPept accession number of invertebrate LHs are listed in Supplementary Table 2.

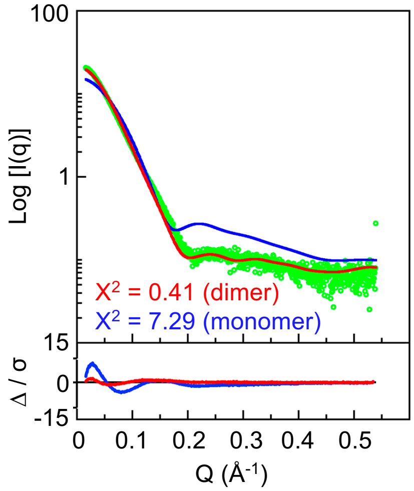

Supplementary Figure 4 │ L230 forms a dimer in solution. Small angle X ray scattering (SAXS) analysis. Top, Curves modeling monomeric (blue) and dimeric (red) L230 LH domain were fit to experimental SAXS data (green). Bottom, Residual difference plots ($\Delta/\sigma = \frac{I_{exp}(q) - I_{mod}(q)}{\sigma(q)}$) show I(q) is significantly flat on dimer (red) compared to monomer (blue) fit.

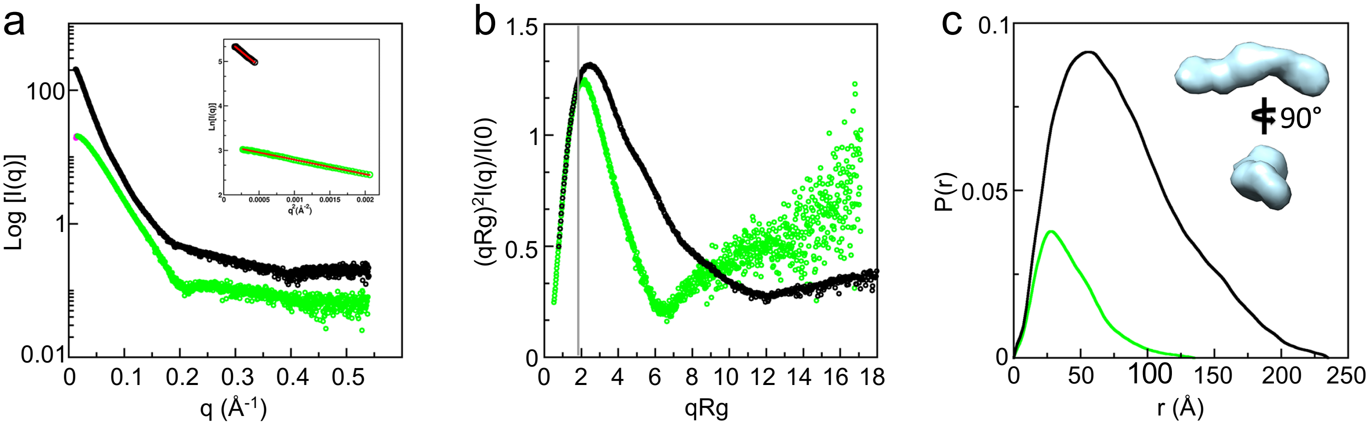

Supplementary Figure 5 │Comparison of L230 and human LH2 structural features. (a) SAXS analysis of full-length LH2 (black) and L230 LH domain (green) with the inlet Guinier plot shows the Guinier fits for qR_g < 1.3. (b) Dimensionless Kratky plot shows that full-length LH2 (black) is relatively more flexible than L230 LH domain (green). The most globular compact protein should have peak at qR_g=√3=1.732 (vertical gray line). The peak shifts to the right of qR_g > 1.732 meaning that it is more flexible or elongated. (c) Pair-distribution function shows that full-length LH2 (black) and L230 LH domain (green) both have an elongated shape (SAXS envelope of LH2 shown in cyan), supporting a tail-to-tail dimerization.

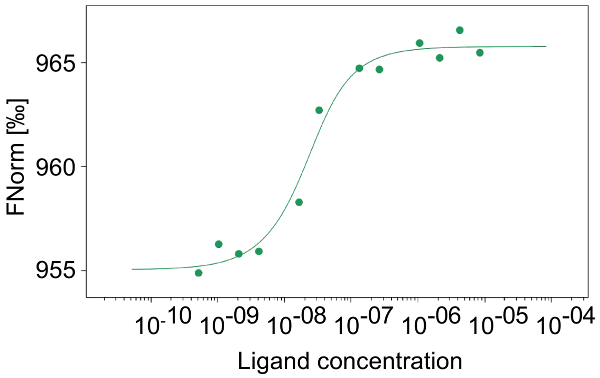

Supplementary Figure 6 │ Dimer affinity of the L230 LH domain. Microscale thermophoresis was used to determine the K_d value for L230 dimer (23.9 ± 2.4 nM).

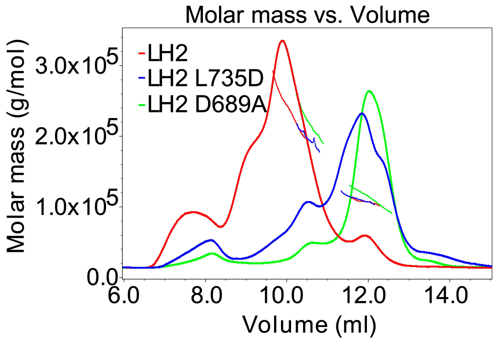

Supplementary Figure 7 │ Human LH2 dimer assemblies require the conserved leucine and Fe^{2+}. SEC-MALS was performed on full-length human LH2 that is wild-type (LH2) or lacks the conserved leucine (L735D) or is deficient in Fe^{2+} binding (D689A). On the basis of elution volume (X axis) and molar mass (Y axis), the L735D and D689A mutants are monomeric proteins.

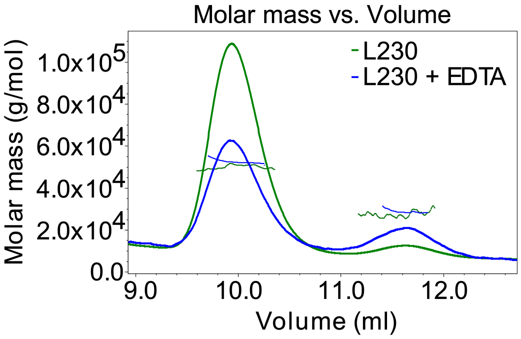

Supplementary Figure 8 │ Dimerization is lost following Fe^{2+} chelation. SEC-MALS was performed on L230 LH domain treated with (L230 + EDTA) or without (L230) 25 mM EDTA. On the basis of elution volume (X axis) and molar mass (Y axis), a portion of EDTA-treated protein is monomeric.

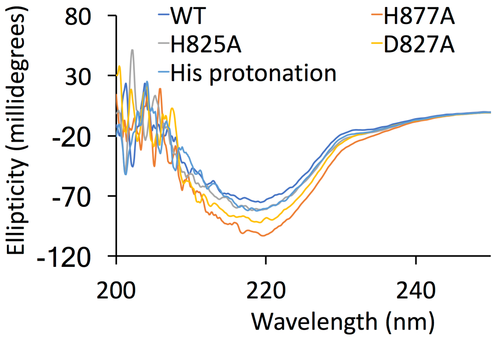

Supplementary Figure 9 │ No alteration in L230 secondary structure due to loss of Fe^{2+}-binding. Circular dichroism spectrometry of wild-type L230 (WT) and L230 that is Fe^{2+}-deficient due to histidine protonation or mutations in the amino acid triad. The proteins demonstrated similar spectra.

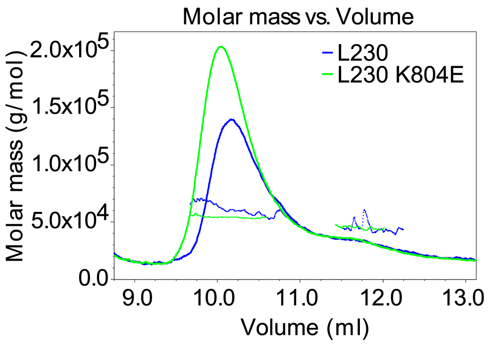

Supplementary Figure 10 │ A cleft mutation does not disrupt dimerization. SEC-MALS was performed on L230 LH domain that is wild-type (L230) or mutant (L230 K804E). On the basis of elution volume (X axis) and molar mass (Y axis), K804E is a dimeric protein.

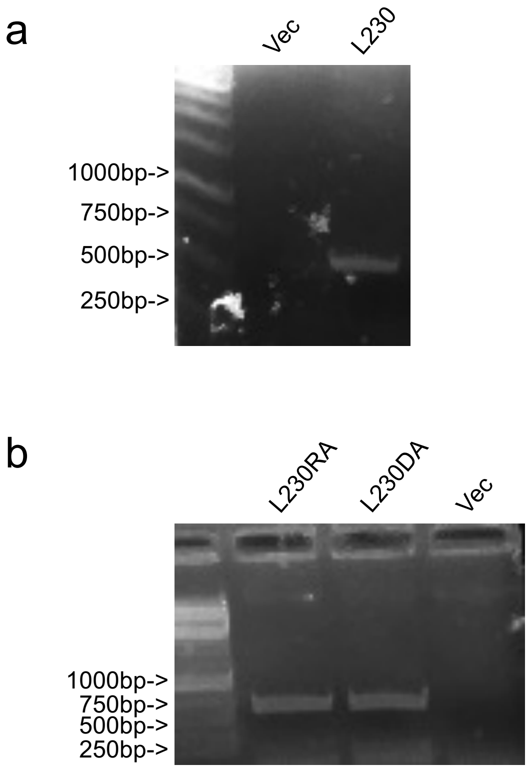

Supplementary Figure 11 | **(a, b)** The ectopic expression of wild-type L230 (a) and mutant L230 (b) in H1299 cells transfected with L230 or empty vector (Vec) was confirmed by PCR. The mutants include L230 D827A (DA) and L230 R887A (RA).

Supplementary Table 1 UniProtKB accession number of LH sequences used in **Extended Data Figure 1b**

Gene name	Species	Protein name	UniProtKB accession number
MIMI_L230	Acanthamoeba polyphaga mimivirus	Procollagen lysyl hydroxylase and glycosyltransferase	Q5UQC3
PLOD1	Homo sapiens	Procollagen-lysine,2-oxoglutarate 5-dioxygenase 1	Q02809
PLOD2	Homo sapiens	Procollagen-lysine,2-oxoglutarate 5-dioxygenase 2	O00469-2
PLOD3	Homo sapiens	Procollagen-lysine,2-oxoglutarate 5-dioxygenase 3	O60568
PLOD1	Danio rerio	Procollagen-lysine,2-oxoglutarate 5-dioxygenase 1	A0JMD1
PLOD2	Danio rerio	Procollagen-lysine,2-oxoglutarate 5-dioxygenase 2	A4U7F9
PLOD3	Danio rerio	Procollagen-lysine,2-oxoglutarate 5-dioxygenase 3	Q1L618
PLOD	Drosophila melanogaster	Procollagen lysyl hydroxylase, isoform A	Q9VTH0
Let-268	Caenorhabditis elegans	Procollagen-lysine,2-oxoglutarate 5-dioxygenase	Q20679

Supplementary Table 2 Invertebrate LH NCBI GenPept accession numbers

Species	NCBI GenPept accession number
Amphimedon queenslandica	XP_019854114.1
Trichoplax adhaerens	XP_002113369.1
Hydra vulgaris	XP_002157097.1
Strongylocentrotus purpuratus	XP_011661533.1
Saccoglossus kowalevskii	XP_006811878.1
Ciona intestinalis	XP_002131164.1
Branchiostoma belcheri	XP_019623603.1
Rhodnius neglectus	JAI55597.1
Hypsibius dujardini	OQV13852.1
**Drosophila melanogaster**	NP_648451.1
Tetranychus urticae	XP_015786267.1
Lingula anatina	XP_013392392.1
Daphnia magna	JAN34138.1
Crassostrea gigas	XP_011437798.1
Lottia gigantea	XP_009053597.1
Octopus bimaculoides	XP_014782723.1
Orchesella cincta	ODM93852.1
Trichuris trichiura	CDW52628.1
Trichinella pseudospiralis	KRY65651.1
**Caenorhabditis elegans**	NP_496170.1
Capitella teleta	ELT92140.1
Helobdella robusta	XP_009020625.1
Echinococcus multilocularis	CDS37198.1
Oikopleura dioica	CBY36038.1
Hormiphora californensis	AQX17749.1
Beroe abyssicola	AQX17751.1
Euplokamis dunlapae	AQX17750.1
Intoshia linei	OAF69316.1

Supplementary Table 3 Summary of data analysis of the experimental SAXS profiles

**Data Collection**	L230	LH2
Beamline	ALS Beamline BL12.3.1	ALS Beamline BL12.3.1
Beam energy (keV)	11	11
Sample-detector distance (m)	1.5	1.5
Detector	Pilatus	Pilatus
Exposure time (s)	Every 0.3s. Total: 10.2 s.	Every 0.3s. Total: 10.2 s.
Images	Total: 33 images	Total: 33 images
Sample cell thickness (mm)	1.5	1.5
Temperature (K)	283	283
Final q range (Å^{-1})	0.01 to 0.5	0.01 to 0.5
**Data Analysis**		
Programs	SCÅTTER 3.0	SCÅTTER 3.0
Buffer	20 mM Tris, pH 8, 200 mM NaCl	20mM Tris, pH 8, 200mM NaCl, 1% Glycerol
Protein concentration (mg/ml)	1.5, 3, 6	1, 3, 5
Points used for Guinier analysis	7-52	1-16
Guinier qR_g limits	1.3	1.3
$I(0)$ (cm^{-1})	22.830 ± 0.038	257.000 ± 1.466
Guinier R_g (Å)	31.82 ± 0.26	62.85 ± 2.26
D_{max} (Å)	135	235
R_g (real) (Å)	33.78 ± 0.712	65.28 ± 0.307
R_g (reciprocal) (Å)	33.59	64.93
Ab initio Modeling	DAMMIF	GASBOR
MW estimation (Vc based) (kDa)[1]	41	200
Porod Volume (Å^3) (q-range (Å^{-1}))	105863 (0.01 – 0.140)	500680 (0.01 – 0.120)
Porod Exponent	3.7	3.6

[1] MW is estimated based on $MW = \frac{(V_c)2/R_g}{0.1231}$, using SCÅTTER program.

Supplementary Table 4 MW of L230 and human LH2 proteins determined by SEC-MALS

Conditions*	$MW_{monomer}$ (kDa)	Mass fraction (%)	MW_{dimer} (kDa)	Mass fraction (%)
L230	28.8 (± 2.0%)	10.5	52.6 (± 2.5%)	89.5
L230 H825A	35.1 (± 0.9%)	94.1	-	-
L230 D827A	29.9 (± 2.4%)	90.6	53.5 (± 7.9%)	9.4
L230 A869G	28.4 (± 1.7%)	12.2	51.3 (± 2.7%)	87.8
L230 L873D	28.5 (± 2.8%)	94.9	51.2 (± 2.3%)	5.1
L230 H877A	28.6 (± 1.3%)	92.9	55.6 (± 1.4%)	7.1
L230 R887A	26.5 (± 2.1%)	8.8	50.4 (± 3.7%)	91.2
L230 + EDTA	37.0 (± 4.3%)	34.5	58.8 (± 2.0%)	65.5
L230/His protonation	26.3 (± 1.6%)	79.4	52.5 (± 2.8%)	20.6
L230/His protonation + Fe^{2+} reconstitution	26.7 (± 2.5%)	24.5	51.5 (± 1.6%)	75.5
LH2	107.4 (± 1.2%)	21.8	239.2 (± 0.2%)	78.2
LH2 L735D	111.2 (± 3.5%)	84.8	196.7 (± 3.2%)	15.2
LH2 D689A	110.3 (± 0.9%)	95.0	215.9 (± 1.2%)	5.0

* L230 includes LH domain only (aa 680-895). LH2 includes aa 33-758.

Supplementary Table 5 DNA primers used in this study

Name	Primer sequence
L230 forward1	GTGCTGCCCGGGGCCACCATGGGGGGATGCACGGTG
L230 forward2	GAGACCTCGAGGCAGCGTTCCTACCGAAGTTACTCTTTATGATCTTCC
L230 reverse	GCGTCGGCGGCCGCTTAATTAACAAAAGACACTAAAATATATCTTTTACCGGAAGTAATTGG
D827A	GACAATCTGAATTGGCTCCTCATCATGCTTCTTCCACATATACTTTAAATATTGCACTTAATG
L873D forward	GTAATTGGAAGAGCTCGATGATATGCCAAGTCTTTTCCAGCGTGAATTGTAGCGTAACC
L873D reverse	GGTTACGCTACAATTCACGCTGGAAAAGACTTGGCATATCATCGAGCTCTTCCAATTAC
H877A forward	CACGCTGGAAAACTATTGGCATATGCTCGAGCTCTTCCAATTACTTCCGG
H877A reverse	CCGGAAGTAATTGGAAGAGCTCGAGCATATGCCAATAGTTTTCCAGCGTG
H825A forward	GCAATATTTAAAGTATATGTGGAAGAATCATGAGCAGGAGCCAATTCAGATTGTCTTTCCATATC
H825A reverse	GATATGGAAAGACAATCTGAATTGGCTCCTGCTCATGATTCTTCCACATATACTTTAAATATTGC
R887A forward	GGCCGCTTAATTAACAAAAGACACTAAAATATATGCTTTACCGGAAGTAATTGGAAGAGCTCG
R887A reverse	CGAGCTCTTCCAATTACTTCCGGTAAAGCATATATTTTAGTGTCTTTTGTTAATTAAGCGGCC
K804E forward	CGTCATTTATACAATAATTATAAAACCGAAGATATTAATTTAGCTTTTGTTGTTAAATATG
K804E reverse	CATATTTAACAACAAAAGCTAAATTAATATCTTCGGTTTTATAATTATTGTATAAATGACG
A881G forward	GGAAAACTATTGGCATATCATCGAGGTCTTCCAATTACTTCCGGTAAAAG
A881G reverse	CTTTTACCGGAAGTAATTGGAAGACCTCGATGATATGCCAATAGTTTTCC
LH2 forward	GCGTCGTCTAGAAGCATCCCCACAGATAAATTATTAGTCATAACTGTAG
LH2 reverse	GCGTCGGCGGCCGCTCAGGGATCTATAAATGACACTGCAATGTATCTTGTTCC
LH2L735D forward	GGAGCTTCATGCATCCTGGGAGAGACACACATTTGCATGAAGGACTTCCTG
LH2L735D reverse	CAGGAAGTCCTTCATGCAAATGTGTGTCTCTCCCAGGATGCATGAAGCTCC

Supplementary Table 6 Data collection and structure refinement statistics

	L230_Iodine	L230_Native
**Data collection**		
Beamline	GM/CA–CAT 23-ID-D	GM/CA–CAT 23-ID-B
Wavelength (Å)	1.77	0.98
Space group	$P4_32_12$	$P4_32_12$
Cell dimensions		
a, b, c (Å)	109.21, 109.21, 83.91	109.46 109.46 84.02
Number of reflections measured	577481	169899
Number of unique reflections	24748	34800
Resolution (Å)*	28.41-2.24 (2.32-2.24)	48.95-2.00 (2.07-2.00)
R_{merge}	0.117 (0.759)	0.160 (1.03)
R_{pim}	0.0234 (0.311)	0.0793 (0.516)
$CC_{1/2}$	0.999 (0.635)	0.993 (0.507)
Mean I/sigma(I)	23.95 (1.80)	8.82 (1.44)
Completeness (%)	97.65 (84.19)	99.61 (99.56)
Redundancy	23.3 (6.4)	4.9 (4.9)
**Refinement**		
Resolution (Å)	28.49-2.24	
Number of reflections used in refinement	24380	34795
Number of reflections used for R-free	1961	1753
R_{work}/R_{free}	0.223/0.241	0.183/0.216
Number of atoms		
All	3315	3718
Protein	3171	3271
Ligand/ion	102	2
Water	42	445
B-factors		
Protein	34.64	27.27
Ligand/ion	77.83	44.04
Water	40.6	37.29
R.m.s. deviations		
Bond lengths (Å)	0.002	0.002
Bond angles (º)	0.5	0.52
Ramachandran statistics		
Favored regions (%)	95.21	93.93
Allowed regions (%)	4.79	6.07
Outliers (%)	0	0

* Highest resolution shell is shown in parentheses.
